# Residency Training at the Front of the West African Ebola Outbreak: Adapting for a Rare Opportunity

**DOI:** 10.1371/currents.outbreaks.2ccbcab30e96d3fe28d3896d258b818e

**Published:** 2016-02-02

**Authors:** Yin Mo, Sophia Archuleta, Sharon Salmon, Dale Fisher

**Affiliations:** Ministry of Health Holdings, Singapore; Division of Infectious Diseases, University Medicine Cluster, National University Health System, Singapore; Division of Infectious Diseases, University Medicine Cluster, National University Health System, Singapore; Yong Loo Lin School of Medicine, National University of Singapore, Singapore; Department of Nursing, National University Health System, Singapore; Division of Infectious Diseases, University Medicine Cluster, National University Health System, Singapore; Yong Loo Lin School of Medicine, National University of Singapore, Singapore

## Abstract

Medical trainees face multiple barriers to participation in major outbreak responses such as that required for Ebola Virus Disease through 2014-2015 in West Africa. Hurdles include fear of contracting and importing the disease, residency requirements, scheduling conflicts, family obligations and lack of experience and maturity. We describe the successful four-week deployment to Liberia of a first year infectious diseases trainee through the mechanism of the Global Outbreak Alert and Response Network of the World Health Organization. The posting received prospective approval from the residency supervisory committees and employing hospital management and was designed with components fulfilling the Accreditation Council for Graduate Medical Education (ACGME) core competencies. It mirrored conventional training with regards to learning objectives, supervisory framework and assessment methods. Together with Centers for Disease Control and Prevention and many other partners, the team joined the infection prevention and control efforts in Monrovia. Contributions were made to a 'ring fencing' infection control approach that was being introduced, including enhancement of triage, training and providing supplies in high priority health-care facilities in the capital and border zones. In addition the fellow produced an electronic database that enabled monitoring infection control standards in health facilities. This successful elective posting illustrates that quality training can be achieved, even in the most challenging environments, with support from the pedagogic and sponsoring institutions. Such experiential learning opportunities benefit both the outbreak response and the trainee, and if scaled up would contribute towards building a global health emergency workforce. More should be done from residency accreditation bodies in facilitating postings in outbreak settings.

## Commentary

Recent publications describe barriers faced by medical trainees in volunteering for the Ebola outbreak in West Africa^1^. Concerns include fear of contracting and importing the disease, violating residency requirements, scheduling conflicts, family obligations, and lack of experience and maturity. There was a theme of doubt over whether the effort and risks justified benefits to the trainee and the overall outbreak response. We describe the successful four-week deployment to Liberia of a first year infectious diseases trainee via the Global Outbreak Alert and Response Network (GOARN) of the World Health Organisation (WHO), which received prospective approval from the residency supervisory committees and employing hospital management.

International electives, despite being widely recognized for their theoretical benefits in residency training, are often entwined with problems in practice^2^. Training bodies must reconcile accreditation requirements with unique educational opportunities and ensure that the quality of training is not limited by poor supervision and demands to practice outside one’s competencies. Employers who fund trainees need to justify the expense with little direct return. There are logistic and safety issues for which someone must assume responsibility. An elective in a massive outbreak such as that in West Africa exacerbated all these concerns. Once overcome, however, the benefits would be arguably all the greater.

A Singapore team consisting of an infectious disease physician and an infection prevention and control expert had been deployed through GOARN to Liberia twice in 2014. The possibility of including a fellow with an interest in this type of work in a third deployment was raised. A program was designed with components mirroring an on-site posting.

Post-graduate medical education in Singapore gained accreditation by the Accreditation Council for Graduate Medical Education-International (ACGME-I) in 2010. Hence, the learning objectives (Fig. 1), supervisory framework and assessment methods of the program aligned closely with the ACGME core competencies. Formal pre-deployment training consisted of a specific course in Darwin in December 2014, co-organised by GOARN and RedR, Australia. In addition, specific prerequisites were met including the UN-mandated online safety course and health requirements.

Concurrently, an application proposal was submitted to the national residency training committees and the sponsoring institution. This brought about concerns similar to those previously cited: safety, residency requirements, and the value of learning, especially during the first year of training. With justifications the elective received approval. Deployment costs of the posting were met by GOARN while the wage was maintained locally.

In February 2015, the Singapore team of three joined the Liberian infection prevention and control efforts in Monrovia. A 'ring fencing' infection control approach was being introduced, including enhancement of triage, training and providing supplies in high priority health-care facilities in the capital city and at the nation’s borders^3^. The fellow produced an electronic database that enabled monitoring infection control standards in the health facilities. This aided the Liberian Ministry of Health in prioritizing resources and united efforts from the various NGOs to improve efficiency and accountability. During this period, there were close working relationships with the many partners also assisting in the response.

Throughout the month, there was continuous supervision and mentoring with time allocated for reflection. Regular feedback sessions focused on providing constructive appraisals and encouraging self-directed learning. Assessment methods emphasized the competencies and included multi-source assessment and structured discussions. The final evaluation was based on the concluding presentation of the team’s achievements to the WHO Representative for Liberia, provided in part by the fellow.

The volatile outbreak situation gave rise to a number of difficulties that challenged the team in adhering to the requirements of the rotation. Long working hours would have been considered a violation of the ACGME duty hours. The outbreak setting constantly challenges one’s skills, both technical and interpersonal. An example is the need to negotiate with local health managers and partners to align everyone’s efforts and work with the inefficiencies inherent in resource-limited settings. However, these seemingly uncomfortable circumstances strengthened the experience in providing a holistic perspective in problem solving.

The team’s work received commendations from both the WHO Country Office, Liberia and local leaderships. Upon return, the experience was shared with audiences including medical students, residents and senior doctors, nurses, and staff of the Singapore Ministry of Health. Stories from the field were widely deemed to add value to the Singaporean preparedness efforts.

The posting fulfilled all criteria previously proposed for international health electives^4^ ^3^.The fellow provided meaningful service to the host nation and this was relevant in the trainee’s home setting. The posting helped the trainee in analyzing her career aspirations and becoming a focal point by sharing the experiences with future aspirants. In 2010, the Working Group on Ethics Guidelines for Global Health Training provided advice for institutions and trainees on ethics and best practices^5^ ^4^. This elective conformed to the guidelines which are appropriate and intuitive. A structured program was developed and adhered to so that "host and sender as well as other stakeholders derived mutual, equitable benefits".

There is strong evidence of benefits of global health training across the ACGME core competencies^6^ ^5^. Despite the high level of interest from learners, there is no formal registry of residency programs in the US or anywhere to our knowledge that offers formalized global health electives as part of a training curriculum.

Infectious disease is a unique domain in medicine. Its far-reaching scope disregards geographical and socioeconomic divides. In recent decades, public health emergencies have become larger in scale following closer commercial ties and exponential growth in international travel. Infection control and outbreak management at a global level, therefore, should have greater emphasis in infectious disease training. Increased opportunities for hands-on experience is an ideal mechanism. Conventional residency requirements are limited to the theories of infection control and outbreak investigations, and methods of evaluation are generally multiple-choice questions and discussions on hypothetical scenarios. Beyond gaining technical knowledge, participating in a public health outbreak response instills important skills in leadership, including adaptability to complex and evolving situations, working with people of various backgrounds and expectations, communications and dealing with team dynamics.

The benefits of such an initiative go beyond training however. A youthful inexperienced fellow adds diversity to an outbreak response team and with that comes “fresh eyes”, new perspectives and indeed some skills beyond the experienced campaigner. They are well versed with information technology, social networking capacities and other web-based platforms, which may provide practical solutions in communications, data sharing, and maintenance of key indicators especially in resource-poor settings. Trainees may also better connect with ground staff which can be used to the team’s advantage in implementation of policies and soliciting feedback.

In the wake of the response to the Ebola outbreak, the WHO reform agenda includes six key items, one of which is the development of a global health emergency workforce^7^ ^6^. Indeed, with this proclaimed proactive, longer term view, it would seem remiss to not engage enthusiastic trainees who are still deciding a career path.

Outbreak response skills in infectious disease physicians are required globally but residency accreditation bodies have yet to recognize their role in facilitating supervised experiential learning. Residency training needs to be more flexible in its scheduling and supervision requirements. Outbreaks are unpredictable in time and location. Residents should be allowed to shuffle their postings to apply for a position in outbreak investigation teams or organizations. Faculty supervisors in this setting can be a short-term appointment with the condition of their adherence to training requirements including appropriate guidance, teaching and evaluation. Not every outbreak is a suitable learning opportunity. A set of criteria, weighing the potential systemic and personal benefits and risks, can be drawn up to evaluate each outbreak for training. This can aid the training accreditation bodies and sponsoring institutions to come to a consensus.

This successful elective posting illustrated how quality training was achieved, even in this most challenging environment. Residency accreditation bodies could adapt to encourage and facilitate such postings which in addition benefits the overall outbreak response and contributes towards building a future global health emergency workforce.

**Meeting residency educational requirements during Ebola outbreak deployment: framework of competency-based goals and objectives and assessment methods. *There was no direct patient contact.**
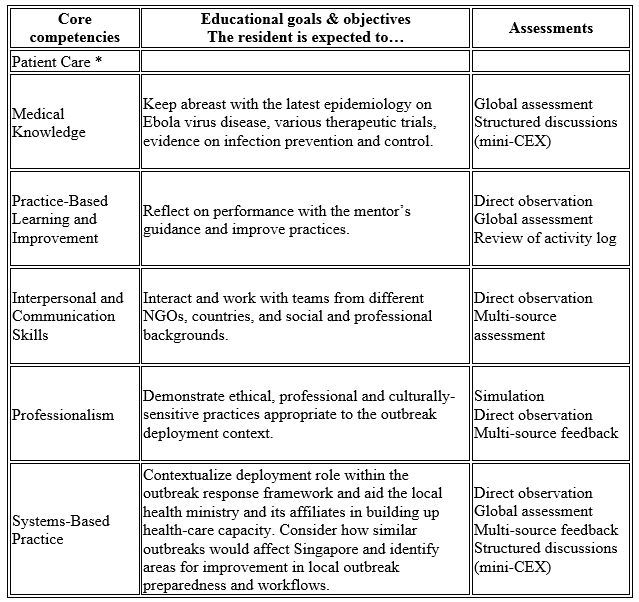

## Competing Interest

The authors have declared that no competing interests exist.

## References

[1] Lisa Rosenbaum. License to serve — U.S. Trainees and the Ebola epidemic. N Engl J Med 2015; 372:504-506 10.1056/NEJMp1415192 25517575

[2] Drain PK, Holmes KK, Skeff KM, Hall TL, Gardner P. Global health training and international clinical rotations during residency: current status, needs, and opportunities. Academic medicine : journal of the Association of American Medical Colleges. 2009; 84:320-325 10.1097/ACM.0b013e3181970a37 PMC3998377 19240438

[3] Cooper C, Fisher D, Gupta N, MaCauley R, Pessoa-Silva CL. Infection prevention and control of the Ebola outbreak in Liberia, 2014-2015: key challenges and successes. BMC Med. 2016;14(1):2 10.1186/s12916-015-0548-4 PMC4702360 26732586

[4] Sidney Coupet, John Del Valle. A case for an international health elective training program during residency: a four­points call for action. Teaching and Learning in Medicine, an international journal 2013; 25:266­271 10.1080/10401334.2013.797347 23848335

[5] John A. Crump, Jeremy Sugarman, Working Group on Ethics Guidelines for Global Health Training (WEIGHT). Global health training: ethics and best practice guidelines for training experiences in global health. Am J Trop Med Hyg 2010; 83:1178–1182 10.4269/ajtmh.2010.10-0527 PMC2990028 21118918

[6] Gladding, Sophia et al. International electives at the University of Minnesota global pediatric residency program: opportunities for education in all accreditation council for graduate medical education competencies. Academic Pediatrics 2012; 12:245 - 250 10.1016/j.acap.2012.02.009 22483843

[7] WHO, WHO emergency reform milestones: Face-to-face meeting of the Advisory Group on Reform of WHO’s work in outbreaks and emergencies with Health and Humanitarian Consequences, 26- 27 October 2015

